# Supplementary material for: Content-rich biological network constructed by mining PubMed abstracts
Source: BMC Bioinformatics. 2004 Oct 8;5:147. doi: 10.1186/1471-2105-5-147 (PMC528731; doi:10.1186/1471-2105-5-147)
Supplement: Additional File 2 — The original results of the above study (non-essential files are deleted to keep the file size under the limit set by BMC bioinformatics). [file 1471-2105-5-147-S2.bz2 › chilibotAdditionalFile2/dip05/757ID11872715E18143/html/OMPA_TONB.html]

 


 **OMPA** and **TONB** 
  
Found 7 abstracts in PubMed, retrieved 05.  
 

 What does Google say? 
 PDF only 
| .edu only 

---

**Interactive relationship** (e.g. stimulation, inhibition, etc)

**Inhibitory relationship**- The coupling of FepA L7 to OmpF, OmpC, or  **OmpA**  was  **TonB**  independent and was unaffected by the uncouplers CCCP carbonyl cyanide m chlorophenylhydrazone and DNP dinitrophenol but completely inhibited by cyanide.  Ref: 12169616 J Bacteriol, 2002
**Neutral relationship**- N terminal amino acid sequence analysis revealed that major outer membrane proteins in this organism included Arg gingipain, Lys gingipain, RagA a  **TonB**  linked receptor, and putative porins that were homologous to Escherichia coli  **OmpA** .  Ref: 12013560 Eur J Oral Sci, 2002

**Non-interactive relationship** (e.g. studied together, co-existance, homology, etc.)

- The identifications include several  **TonB**  dependent receptors, two  **OmpA**  family proteins, a dipeptidase, GlpQ, two alkaline phosphatases, 3 phytase, a putative TolC protein and 11 proteins of unknown function.  Ref: 12180922 Mol Microbiol, 2002
- Subsequently, two additional contacts of  **TonB**  with the outer membrane proteins Lpp and, putatively,  **OmpA**  were identified by in vivo cross linking.  Ref: 11872715 J Bacteriol, 2002
- They include the  **OmpA**  membrane domain, the OmpX protein, phospholipase A, general porins OmpF, PhoE, substrate specific porins LamB, ScrY and the  **TonB**  dependent iron siderophore transporters FhuA and FepA.  Ref: 10931321 Mol Microbiol, 2000
